# Growth habit and leaf economics determine gas exchange responses to high elevation in an evergreen tree, a deciduous shrub and a herbaceous annual

**DOI:** 10.1093/aobpla/plv115

**Published:** 2015-10-03

**Authors:** Zuomin Shi, Matthew Haworth, Qiuhong Feng, Ruimei Cheng, Mauro Centritto

**Affiliations:** 1 Institute of Forest Ecology, Environment and Protection, Key Laboratory on Forest Ecology and Environmental Sciences of State Forestry Administration, Chinese Academy of Forestry, Beijing 100091, China; 2 Trees and Timber Institute, National Research Council, Via Madonna del Piano 10, 50019 Sesto Fiorentino (FI), Italy; 3 Sichuan Academy of Forestry, Chengdu 610081, China

**Keywords:** Elevation, mesophyll conductance, partial pressure of CO_2_, photosynthesis, *Quercus spinosa*, *Rumex dentatus*, *Salix atopantha*, stomatal conductance

## Abstract

Plant growth at high elevations necessitates physiological and morphological plasticity to enable photosynthesis (*A*) under conditions of reduced temperature, increased radiation and the lower partial pressure of atmospheric gases, in particular carbon dioxide (*p*CO_2_). Previous studies have observed a wide range of responses to elevation in plant species depending on their adaptation to temperature, elevational range and growth habit. Here, we investigated the effect of an increase in elevation from 2500 to 3500 m above sea level (a.s.l.) on three montane species with contrasting growth habits and leaf economic strategies. While all of the species showed identical increases in foliar δ^13^C, dark respiration and nitrogen concentration with elevation, contrasting leaf gas exchange and photosynthetic responses were observed between species with different leaf economic strategies. The deciduous shrub *Salix atopantha* and annual herb *Rumex dentatus* exhibited increased stomatal (*G*_s_) and mesophyll (*G*_m_) conductance and enhanced photosynthetic capacity at the higher elevation. However, evergreen *Quercus spinosa* displayed reduced conductance to CO_2_ that coincided with lower levels of photosynthetic carbon fixation at 3500 m a.s.l. The lower *G*_s_ and *G*_m_ values of evergreen species at higher elevations currently constrains their rates of *A*. Future rises in the atmospheric concentration of CO_2_ ([CO_2_]) will likely predominantly affect evergreen species with lower specific leaf areas (SLAs) and levels of *G*_m_ rather than deciduous species with higher SLA and *G*_m_ values. We argue that climate change may affect plant species that compose high-elevation ecosystems differently depending on phenotypic plasticity and adaptive traits affecting leaf economics, as rising [CO_2_] is likely to benefit evergreen species with thick sclerophyllous leaves.

## Abstract (precis)

Elevational changes in temperature and carbon dioxide can be used to predict the likely responses of plants to climate change. However, plant species often exhibit contrasting photosynthetic and morphological responses to altitude. We investigated the photosynthetic responses of plants with different leaf morphologies and growth habits to increased elevation. At high elevations, plants with schlerophyllous leaves that represent a large structural investment are more likely to be positively affected by rising carbon dioxide than species with a lower investment in foliage.

## Introduction

The adaptational responses of plants to alterations in temperature and the partial pressure of carbon dioxide (*p*CO_2_) along elevational gradients have been used to infer the likely responses of vegetation to climatic changes in the past and future (e.g. Körner 2007; Kouwenberg *et al*. 2007; Bai *et al*. 2015). The partial pressure of all atmospheric gases declines with elevation, reducing the availability of CO_2_ for photosynthesis and oxygen for respiration, while lower temperatures and higher levels of radiation may decrease the activity of photosynthetic and metabolic enzymes (Gale 1972*a*; Miroslavov and Kravkina 1991; Allen and Ort 2001). Growth at high elevation, therefore, necessitates physiological and morphological adaptations to enable photosynthesis (e.g. Woodward 1986; Williams and Black 1993; Terashima *et al*. 1995; Cordell *et al*. 1998; Körner 2007; Feng *et al*. 2013). High-elevation ecosystems are often more sensitive to climatic change than those at sea level, as the influence of rising atmospheric temperature and carbon dioxide concentration ([CO_2_]) on photosynthesis becomes more pronounced with elevation (Gale 1972*a*, *b*, 1973; Terashima *et al*. 1995). Nonetheless, not all plant species respond in the same manner along elevational gradients, and comparatively little is known regarding the physiological and morphological adaptation of plants growing at high elevations >2500 m above sea level (a.s.l.). An understanding of the physiological and morphological processes that underpin photosynthesis and leaf gas exchange at high elevations may assist in our understanding of the likely impacts of future climate change on these high-elevation ecosystems.

In addition to physical changes to *p*CO_2_ and temperature, an increase in elevation may also be associated with fluctuations in soil type, wind speed, water availability and the quality/quantity of incident radiation (Körner 2007). These factors will all influence plant growth and photosynthesis (e.g. Körner and Cochrane 1985; Woodward 1986). The photosynthetic performance of a plant is determined by its capacity for the uptake and assimilation of CO_2_ (Farquhar *et al*. 1980); this is controlled by stomatal (*G*_s_) and mesophyll conductance (*G*_m_) to CO_2_, the carboxylation capacity of ribulose-1,5-bisphosphate (RuBP) carboxylase/oxygenase (RubisCO) (*V*_cmax_) and the maximum rate of electron transport for RuBP regeneration (*J*_max_) (e.g. Shi *et al*. 2006; Centritto *et al*. 2009; Haworth *et al*. 2011; Feng *et al*. 2013). Stomatal conductance frequently increases with elevation, possibly as a result of the lower availability of CO_2_ in the atmosphere necessitating increased rates of conductance to sustain adequate CO_2_ uptake (Körner *et al*. 1986; Woodward 1986). This enhanced *G*_s_ with elevation is often accompanied by increased stomatal density values and higher rates of transpirative water loss (Körner and Cochrane 1985; Woodward and Bazzaz 1988; Kouwenberg *et al*. 2007; Wang *et al*. 2014). The lower partial pressures of gases at high elevations will not only result in more rapid diffusion through the stomatal pore of CO_2_ but also water vapour, which when combined with the generally higher irradiances and drier atmosphere at high elevations will enhance the transpirative costs of growth at high elevation (Gale 1972*a*, *b*, 1973). However, these patterns of increased *G*_s_ are not universal, with species such as *Quercus aquifolioides* (Li *et al*. 2006; Feng *et al*. 2013), *Q. guyavifolia* (Hu *et al*. 2015) and *Typha orientalis* (Bai *et al*. 2015) exhibiting reduced *G*_s_ and stomatal density with elevation. Species with reduced *G*_s_ at high elevation may exploit the more rapid diffusion of CO_2_ at low partial pressures (Gale 1972*a*) by increasing rates of carboxylation of CO_2_ to maintain the diffusion gradient between the internal leaf and external atmosphere (Cordell *et al*. 1998; Shi *et al*. 2006) without incurring the additional transpirative costs associated with increased *G*_s_ and stomatal density (Haworth *et al*. 2015). However, lower temperatures at high elevation may also minimize the effect of increased diffusion of CO_2_ at lower partial pressures during the gaseous phase of CO_2_ transport (Körner 2007).

The concentration of CO_2_ inside the chloroplast envelope is directly related to stomatal and mesophyll conductance to the transport of CO_2_ (Loreto *et al*. 2009; Flexas *et al*. 2013). As a result, *G*_s_ and *G*_m_ often operate synchronously to determine photosynthesis (*A*) as a function of the total conductance to CO_2_ (*G*_tot_) (Centritto *et al*. 2003; Hu *et al*. 2010; Lauteri *et al*. 2014). More rapid diffusion of CO_2_ at lower partial pressures may counteract the effect of reduced availability on gaseous transport of CO_2_ through the stomata to the spongy mesophyll (Gale 1972*a*), but this will not influence the transport of CO_2_ through the aqueous phase to the chloroplast (Loreto *et al*. 1994; Flexas *et al*. 2013). As such, the lower partial pressure of CO_2_ will have a significant impact on *A*, and *G*_m_ will play a significant role in determining the response of a plant species to growth at high elevation. It may be expected that an increase in elevation would induce a rise in *G*_m_ to ensure CO_2_ uptake at lower partial pressures; however, *G*_m_ is determined by a number of physical and biochemical factors (Warren 2007; Niinemets *et al*. 2009) that are also affected by elevation (Körner *et al*. 1979, 1986; Kogami *et al*. 2001; Feng *et al*. 2013). The specific leaf area (SLA) of temperate species tends to decrease with elevation, resulting in leaves with more closely packed cells (Friend and Pomeroy 1970; Friend *et al*. 1989; Li *et al*. 2013; Pan *et al*. 2013; Haworth and Raschi 2014) and lower *G*_m_ values (Kogami *et al*. 2001; Niinemets *et al*. 2009; Feng *et al*. 2013). For example, *Polygonum cuspidatum* exhibited a reduction in SLA and *G*_m_ over an elevation range of 10–2500 m (Kogami *et al*. 2001). This reduction in SLA with elevation is likely a result of increased incident radiation inducing a more compact leaf morphology (Niinemets 2001; Poorter *et al*. 2009). Increased elevation also resulted in a decrease of SLA and *G*_m_ values, but no change in *G*_s_, in the tropical Hawaiian tree species *Meterosideros polymorpha* (Cordell *et al*. 1998). In contrast, the highland species *Buddleja davidii* that grows from 1200 to 3500 m a.s.l. exhibited no alteration of SLA, but a reduction in *G*_s_ alongside an increase in *G*_m_ with elevation that corresponded to enhanced *V*_cmax_ (Shi *et al*. 2006). This indicates the importance of the temperature to which a species is adapted and leaf economic strategy in determining the response to elevation (Hovenden and Vander Schoor 2004).

Photosynthesis rates decline with elevation due to lower partial pressures reducing the availability of CO_2_ (Gale 1972*a*; Körner and Diemer 1987) and diminished temperatures resulting in lower activity of photosynthetic enzymes (Fryer *et al*. 1998). The selective pressures exerted by lower temperatures and *p*CO_2_ may induce increased photosynthetic capacity (i.e. enhanced *V*_cmax_ and *J*_max_) at high elevation to enable sufficient CO_2_ uptake (Cordell *et al*. 1998; Shi *et al*. 2006; Feng *et al*. 2013). At higher elevations, greater *V*_cmax_ and *J*_max_ are associated with increased leaf nitrogen and chlorophyll concentrations, but lower photosynthetic nitrogen use efficiency (PNUE) (Cordell *et al*. 1998; Kogami *et al*. 2001; Feng *et al*. 2013). Increased leaf nitrogen levels at higher elevations also commonly correspond to reduced SLA (Feng *et al*. 2013); conversely at sea level, SLA is often positively related to nitrogen concentration (Poorter *et al*. 1990). At high elevations, many species exhibit a reduction in leaf area (Kouwenberg *et al*. 2007), possibly as a response to increased levels of incident radiation that would increase leaf temperatures and transpiration rates through increased leaf to air vapour pressure deficit (Körner and Cochrane 1985). This shift in leaf area would affect the calculation of SLA (Poorter *et al*. 2009), and suggests that the temperature lapse rate and decline in *p*CO_2_ with elevation, and the selective pressures they exert induce differential adaptational responses to those observed at low elevation, and may therefore have different effects on plants with contrasting growth habits and leaf economic strategies.

Plants growing at high elevations of 2500–3500 m a.s.l. experience relatively low *p*CO_2_ in the range of 27.7–24.6 Pa in comparison with *p*CO_2_ of 38 Pa at sea level. The temperature lapse rate equates to an average decline in temperature of 5.5 °C with every 1000 m gained in elevation (Barry 2013). In this study, we aimed to characterize the physiological and morphological adaptations of three plant species with different growth habits: an evergreen tree (*Q. spinosa*), a deciduous shrub (*Salix atopantha*) and a herbaceous annual (*Rumex dentatus*). The responses to growth at high elevation of the three study species were also compared with those of *Q. aquifolioides* from the same habitat in an earlier investigation by Feng *et al*. (2013). To assess the adaptational responses of these plants to high elevations in the range of 2500–3500 m a.s.l., we conducted leaf gas exchange measurements and sampled leaves in the field to (i) analyse photosynthetic physiology at high elevations, (ii) characterize leaf gas exchange through quantification of stomatal and mesophyll conductance responses to CO_2_ and any diffusional limitation to photosynthesis, (iii) gauge the effect of reduced *p*CO_2_ and temperature on leaf economics and nitrogen concentration and (iv) evaluate the likely effect of future climate change in terms of rising *p*CO_2_ and temperature on mountainous species and ecosystems.

## Methods

### Plant material and study area

Three plant species with contrasting growth habits that grow at high elevations in north-western China were chosen for analysis. *Quercus spinosa* is a 6–10 m tall evergreen tree occurring in mountain regions of South East Asia over an elevation range of 1000–3500 m and over a range of 2000–3500 m in south-western China (Wu *et al*. 2011). *Salix atopantha* is a deciduous shrub 1–2 m in size, specific to western and south-western China occurring in mountainous regions at elevations of 2300–3500 m (Chen-Fu and Skvortsov 1998). *Rumex dentatus* is a 0.3–0.7 m tall annual herb that grows on moist slopes from sea level to high elevations (>3500 m a.s.l.) in Asia, North Africa and Europe (Kumar *et al*. 2005). It grows in mountainous regions at elevations of 1200–3600 m in south-western China (Shi and Ming 1987).

Populations of *Q. spinosa*, *S. atopantha* and *R. dentatus* at elevations of 2400 and 3500 m a.s.l. in the Wolong Reserve (south-eastern Tibetan-Qinghai area, Sichuan Province, China) (32°25′–32°53′N, 104°20′–104°41′E) were studied. The populations from the higher and lower elevations did not experience water stress and received full illumination with no shading. The leaves used for gas exchange measurements and collected for leaf economic traits, carbon isotope and nitrogen concentration analysis were at identical developmental stages (i.e. the youngest fully expanded leaf at the end of a branch). Field work was conducted from July to August 2010.

### Gas exchange and fluorescence measurements

Leaf gas exchange and fluorescence parameters of the central leaf section were simultaneously measured using a LI-6400-40 leaf chamber fluorometer (LI-COR, Inc., Lincoln, NE, USA) equipped with a 2-cm^2^ cuvette. One leaf was analysed from six plants for each species at each elevation. The concentration of atmospheric gases is constant with elevation; rather, it is the partial pressure of those gases that declines as elevation increases. All measurements were conducted at the same concentration of [CO_2_] but at the respective partial pressures of 2500 and 3500 m a.s.l. The LiCor Li6400 contains a barometric pressure sensor that allows for compensation of the effects of changes in partial pressure on measurements over a range of 65–115 kPa with an accuracy of and resolution of 0.002 kPa. Standard atmospheric pressure at sea level is 101.325 kPa, and at 3500 m a.s.l., atmospheric pressure is ∼70 kPa, indicating that our measurements were conducted within the operating range of the instrument. The measurements were made *in situ* between 9:00 and 15:00 at a saturating photosynthetic photon flux density (PPFD) of 1200 μmol m^−2^ s^−1^ for *Q. spinosa*, 1400 μmol m^−2^ s^−1^ for *S. atopantha* and 2000 μmol m^−2^ s^−1^ for *R. dentatus* at a CO_2_ concentration of 380 μmol mol^−1^. The saturating PPFD was determined by response curves of *A* to increasing PAR (Ögren and Sundin 1996). Leaf temperature was set at 25 °C, and the relative humidity in the leaf cuvette ranged between 46 and 50 %. The chlorophyll fluorescence yield (i.e. the quantum yield of photosystem II (PSII) in the light, $\Phi \text{PSII}=\Delta F/F_{m}^{\prime }$) was measured using a saturating pulse of white light (10 000 μmol m^−2^ s^−1^) (Genty *et al*. 1989). Mesophyll conductance to CO_2_ diffusion was calculated using the variable *J* method (Harley *et al*. 1992). As this work was conducted in the field, it was not possible to calibrate electron transport rate under non-photorespiratory conditions; therefore, a standard calibration where *α* = 0.85 and *β* = 0.5 was used in the calculation of *G*_m_ (Gilbert *et al*. 2012; Walker and Cousins 2013). The variable *J* method is sensitive to the estimation of the CO_2_ compensation point to photorespiration (Γ*) and leaf respiration (Gilbert *et al*. 2012). While measurements of dark respiration (*R*_n_) were also made at ambient CO_2_ concentration in the dark on the same leaves, Γ* used in the gas exchange algorithm was calculated from the Rubisco-specific factors of Galmes *et al*. (2005) using the photosynthetic constants of Von Caemmerer (2000) and formulae of Brooks and Farquhar (1985) (*Q. spinosa*: Γ* = 52.513 μmol^−1^ mol; *S. atopantha*: Γ* = 54.145 μmol^−1^ mol and *R. dentatus*: Γ* = 52.512 μmol^−1^ mol). As Γ* is a relatively conservative parameter (Harley *et al*. 1992), we assumed that the Γ* value used in the gas exchange algorithm did not affect the estimation of *G*_m_. To reduce diffusion leaks through the chamber gasket (Flexas *et al*. 2007), a supplementary external chamber gasket composed of the same polymer foam was added to create an interspace between the two gaskets (i.e. a double-gasket design with a 5-mm space separating the internal and external gaskets). Then the CO_2_ and H_2_O gradients between the in-chamber air and pre-chamber air were minimized by feeding the infra-red gas analyse exhaust air into the interspace between the chamber and the pre-chamber gaskets (Rodeghiero *et al*. 2007). Total conductance to CO_2_ (*G*_tot_) was calculated from mesophyll and stomatal conductance to CO_2_ following Loreto *et al*. (1994) as:

(1) $$G_{\mathrm{tot}}=\frac{G_{s}G_{m}}{G_{s}+G_{m}}$$

Light saturated *A*/*P*_i_ response curves were measured at a leaf temperature of 25 °C and a relative humidity in the leaf cuvette of ∼50 % over a range of [CO_2_] values on a minimum of five plants per species at each elevation. To remove the effect of stomatal limitation on *A*, the leaves were first pre-conditioned at a [CO_2_] of 50 µmol mol^−1^ for ∼60 min to force stomatal opening as described by Centritto *et al*. (2003). The concentration of [CO_2_] within the cuvette was then progressively increased to 2000 µmol mol^−1^. The photosynthetic parameters *A*_max_ (net CO_2_ assimilation rate under conditions of PPFD and CO_2_ saturation), *V*_cmax_ (RuBP-saturated rate of Rubisco: estimate of the carboxylation efficiency of Rubisco determined from the slope of the *A*/*P*_i_ curve at a [CO_2_] of 40–200 µmol mol^−1^) and *J*_max_ (maximum rate of electron transport) were estimated by fitting the mechanistic model of Farquhar *et al*. (1980).

### Leaf sampling, carbon isotope discrimination and leaf nitrogen analysis

Immediately after the gas exchange measurements, two leaves per plant from six plants per species at each elevation were detached and stored in sealed plastic bags for the measurement of leaf area, leaf weight, leaf nitrogen concentration and foliar δ^13^C. Leaf area was measured with a Li-3000 leaf area metre (LI-COR, Inc.). The leaves were then dried at 80 °C for 48 h, the dry mass recorded and then ground into a fine powder using a ceramic grinding container. Specific leaf area (cm^2^ g^−1^) was determined as the leaf area to leaf dry mass ratio. Nitrogen concentration (N_mass_, mg g^−1^) was measured on 0.1 g of dried, ground tissue by using standard Kjeldahl technique and assayed for ammonium with an ultraviolet visible spectrophotometer (Tu1221, Beijing Purkinje General Instrument Company, Beijing, China). N_area_ (nitrogen concentration on a leaf area basis, g m^−2^) and PNUE (µmol mol^−1^ s^−1^) (Hikosaka *et al*. 2002) were calculated using the following formulae:

(2) $${\text{N}}_{\mathrm{area}}=\frac{10\times N_{\mathrm{mass}}}{\mathrm{SLA}}$$

(3) $$\text{PNUE}=\frac{A\times 14}{{\text{N}}_{\mathrm{area}}}$$

where 14 is the atomic mass of nitrogen.

Carbon isotope composition (δ^13^C) was measured on 0.001 g of ground dried tissue by using a continuous flow isotope ratio mass spectrometer. Samples were quantitatively combusted in an elemental analyser (Flash-EA 1112, Thermo Electron, Milano, Italy). The CO_2_ obtained was injected into the helium stream of the mass spectrometer (DELTAplus XP, ThermoFinnigan, Bremen, Germany). The ratio of isotopes (*R* = ^13^C/^12^C) was measured and used to calculate δ^13^C referred to the Pee Dee Belemnite standard according to Farquhar and Richards (1984) as follows:

(4) $$\delta^{13}\text{C}=\frac{R_{\mathrm{sample}}}{R_{\mathrm{standard}}}-1$$

### Statistical analysis

A one-way analysis of variance (ANOVA) was used to assess differences in the data collected from the plants growing at 2500 and 3500 m a.s.l. using the software package SPSS 13.5 (SPSS, Chicago, IL, USA), and graphs were prepared using SigmaPlot 11.0 software (Systat Software Inc., San Jose, CA, USA).

### Ethics statement

The field study did not involve endangered or protected species. No specific permissions or permits were required for the analysis and collection of *Q. spinosa*, *S. atopantha* and *R. dentatus* leaves from the Wolong Reserve (32°25′–32°53′N, 104°20′–104°41′E; Tibetan-Qinghai area, Sichuan Province, China). The leaves were collected from public land with the consent of the responsible government agency (the Chinese Academy of Forestry).

## Results

**Figure 1.**
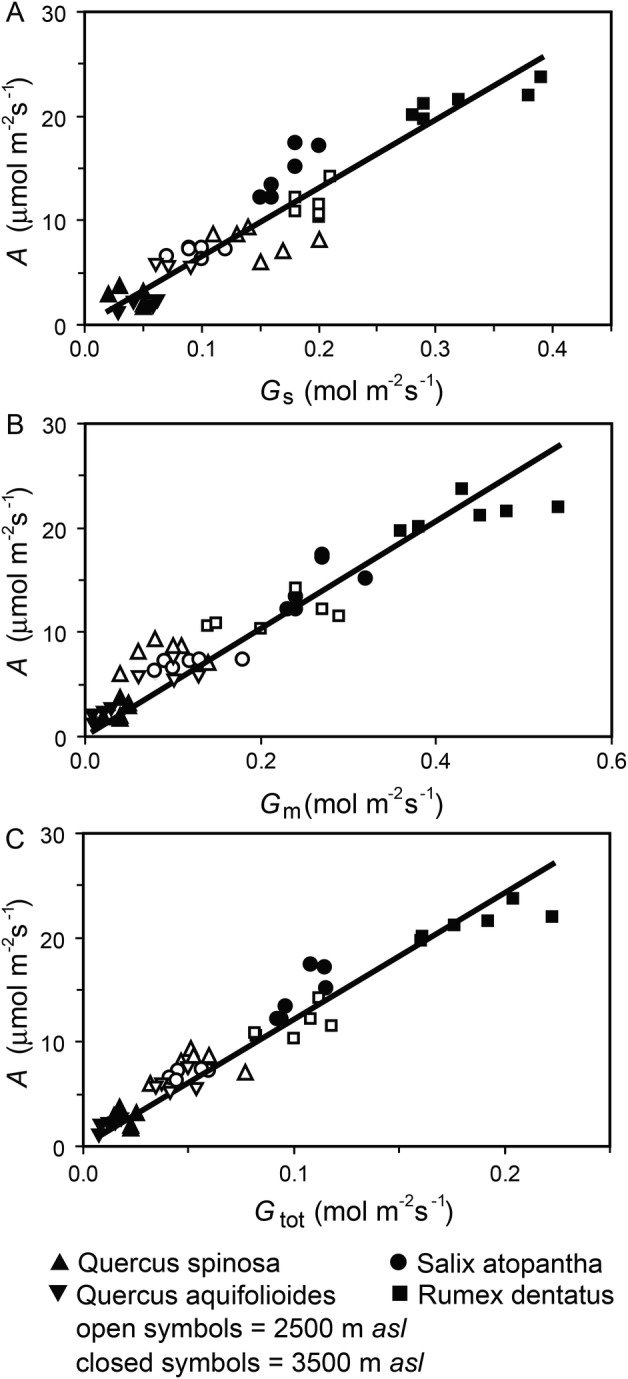
Relationship between photosynthesis (*A*) and stomatal (*G*_s_) (regression *R*^2^ = 0.8836, *F*_1,45_ = 341.775, *P* = 1.197 × 10^−22^), mesophyll (*G*_m_) (regression *R*^2^ = 0.916, *F*_1,45_ = 502.780, *P* = 2.107 × 10^−26^) and total (*G*_tot_) (regression *R*^2^ = 0.943, *F*_1,45_ = 745.674, *P* = 1.195 × 10^−29^) conductance to CO_2_ at high elevations of 2500 m (open symbols) and 3500 m (filled symbols) a.s.l. of *Q. spinosa* (upward triangles), *S. atopantha* (circles) and *R. dentatus* (squares) from this study and *Q. aquifolioides* (inverted triangles) from the study of Feng *et al*. (2013).

**Figure 2.**
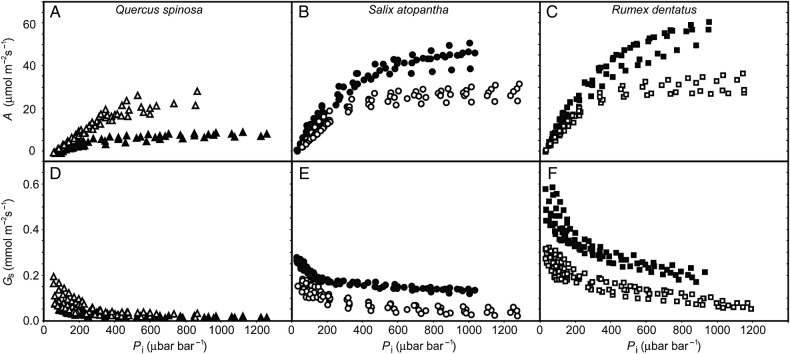
Photosynthetic response curves to internal [CO_2_] (*A/Pi*) and stomatal response to [CO_2_] of plants growing at high elevations of 2500 m (open symbols) and 3500 m (filled symbols) a.s.l. of *Q. spinosa* (A and D), *S. atopantha* (B and E) and *R. dentatus* (C and F). Symbols as in Fig. 1.

Photosynthesis was closely related to conductance to CO_2_ in all of the species at high elevations (Fig. 1). Total conductance (*G*_t_) incorporating both *G*_s_ and *G*_m_ correlated most closely with *A* (Fig. 1C). However, the species analysed did not display identical responses to increased elevation; *Q. spinosa* and *Q. aquifolioides* showed reduced conductance to CO_2_ with an increase in elevation, while *S. atopantha* and *R. dentatus* exhibited higher *G*_s_, *G*_m_ and *G*_tot_ values at higher elevations (Table 1). The annual herb *R. dentatus* and deciduous shrub *S. atopantha* exhibited generally higher values of conductance to CO_2_ and *A* than the evergreen *Quercus* species (Fig. 1). Alterations in *G*_s_ and *G*_m_ with elevation did not significantly affect *P*_i_/*P*_a_ or *P*_c_/*P*_a_ ratios in any of the species analysed (Table 1), suggesting modification of the photosynthetic physiology alongside adjustment in conductance to CO_2_ (Figs 1 and 2). Moreover, the similarity in the ratio of *P*_i_ to *P*_a_ indicates that any variation in or low values of *C*_i_ was unlikely to be responsible for any of the observed patterns in *G*_m_ reported in this study (Tholen *et al*. 2012). Respiration in the dark (*R*_n_) was greater at the higher elevation in all of the species (Table 1). Leaves of *Q. spinosa* exhibited the lowest *R*_n_ values of −1.21 µmol m^−2^ s^−1^ at 2500 m a.s.l., but following a 181.8 % increase showed the highest levels of *R*_n_ at the greater elevation of 3500 m a.s.l., suggesting that the impact of increased elevation was greatest on the species with sclerophyllous evergreen foliage. The two species with leaf lifespans of <9 months, *S. atopantha* and *R. dentatus*, exhibited respective increases of 36.6 and 44.4 % in *R*_n_ between 2500 and 3500 m a.s.l.

**Table 1.** Leaf assimilation rate (*A*), stomatal conductance (*G*_s_), mesophyll conductance (*G*_m_), *P*_i_ (CO_2_ intercellular partial pressure)/*P*_a_ (CO_2_ ambient partial pressure), *P*_c_ (CO_2_ chloroplast partial pressure)/*P*_a_ and *R*_d_ (dark respiration) values of the three plants growing at higher and lower altitudes. Means of a parameter followed by the same letter were not statistically different using a one-way ANOVA (*P* > 0.05) with least significant difference (LSD) *post hoc* test.

	*Q. spinosa*	*S. atopantha*	*R. dentatus*	*F*_5,30_	*P*-value
*A* (μmol m^−2^ s^−1^)					
Low elevation	8.00 ± 0.45 b	7.02 ± 0.17 b	11.62 ± 0.54 c	127.316	2.907 × 10^−19^
High elevation	2.59 ± 0.30 a	14.57 ± 0.88 c	21.33 ± 0.54 d		
*G*_s_ (mol m^−2^ s^−1^)					
Low elevation	0.15 ± 0.01 b	0.10 ± 0.01 c	0.19 ± 0.01 d	79.759	2.096 × 10^−16^
High elevation	0.04 ± 0.01 a	0.17 ± 0.01 d	0.33 ± 0.02 e		
*G*_m_ (mol m^−2^ s^−1^)					
Low elevation	0.09 ± 0.01 a	0.12 ± 0.01 a	0.21 ± 0.02 c	63.573	4.712 × 10^−15^
High elevation	0.04 ± 0.01 b	0.26 ± 0.01 c	0.44 ± 0.02 d		
*R*_n_ (μmol m^−2^ s^−1^)					
Low elevation	1.21 ± 0.04 a	1.34 ± 0.12 ab	1.69 ± 0.14 b	24.353	9.959 × 10^−10^
High elevation	3.41 ± 0.29 e	1.83 ± 0.06 c	2.44 ± 0.12 d		
*P*_i_/*P*_a_					
Low elevation	0.56 ± 0.02 a	0.58 ± 0.01 a	0.65 ± 0.02 b	3.549	0.0122
High elevation	0.58 ± 0.02 a	0.61 ± 0.01 ab	0.65 ± 0.01 b		
*P*_c_/*P*_a_					
Low elevation	0.31 ± 0.02 a	0.35 ± 0.02 ab	0.42 ± 0.03 b	4.444	0.00379
High elevation	0.31 ± 0.03 a	0.36 ± 0.01 a	0.44 ± 0.01 b

**Figure 3.**
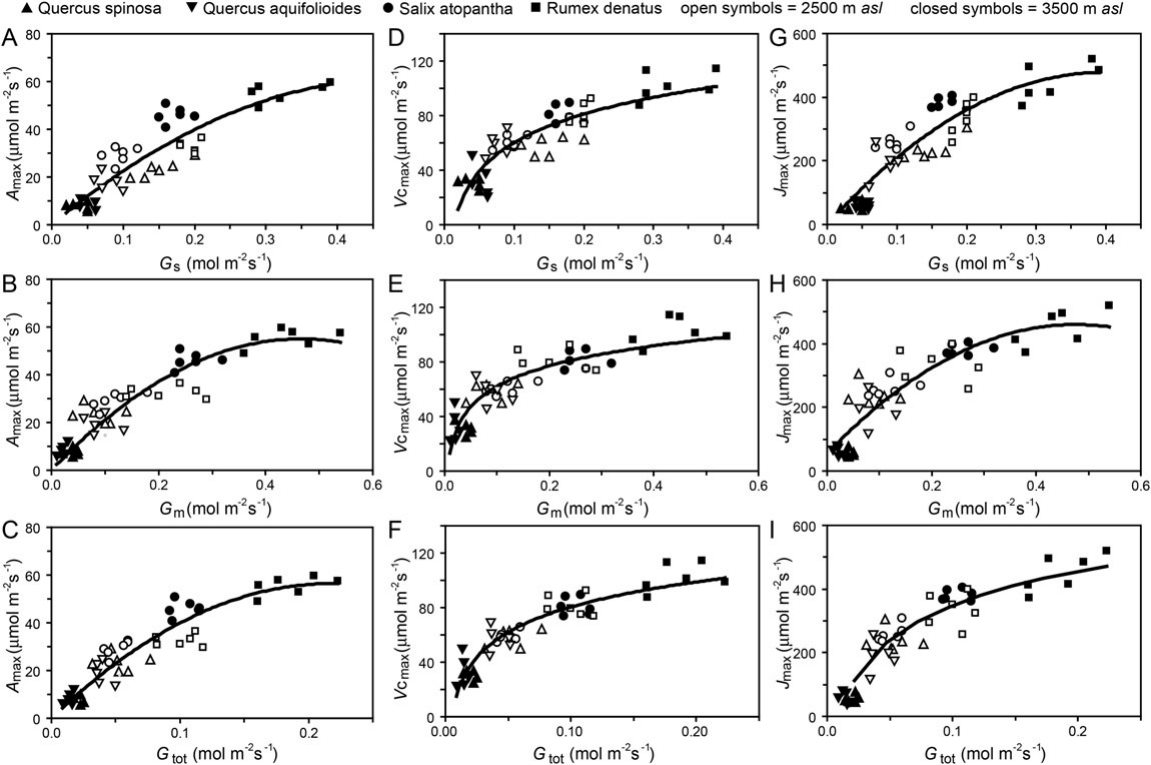
Relationship between photosynthetic physiological capacity to assimilate CO_2_ (*A*_max_, *V*_cmax_ and *J*_max_) and conductance to CO_2_ (*G*_s_, *G*_m_ and *G*_tot_) at high elevations of 2500 m (open symbols) and 3500 m (filled symbols) a.s.l. of *Q. spinosa* (upward triangles), *S. atopantha* (circles) and *R. dentatus* (squares) from this study and *Q. aquifolioides* (inverted triangles) from the study of Feng *et al*. (2013). (A) *A*_max_ versus *G*_s_ (regression *R*^2^ = 0.791, *F*_1,45_ = 147.009, *P* = 1.272 × 10^−15^); (B) *A*_max_ versus *G*_m_ (regression *R*^2^ = 0.847, *F*_1,45_ = 227.498, *P* = 3.250 × 10^−19^); (C) *A*_max_ versus *G*_tot_ (regression *R*^2^ = 0.872, *F*_1,45_ = 223.520, *P* = 7.399 × 10^−19^); (D) *V*_cmax_ versus *G*_s_ (regression *R*^2^ = 0.788, *F*_1,45_ = 156.650, *P* = 4.277 × 10^−16^); (E) *V*_cmax_ versus *G*_m_ (regression *R*^2^ = 0.794, *F*_1,45_ = 141.911, *P* = 1.642 × 10^−15^); (F) *V*_cmax_ versus *G*_tot_ (regression *R*^2^ = 0.859, *F*_1,45_ = 181.645, *P* = 3.186 × 10^−17^); (G) *J*_max_ versus *G*_s_ (regression *R*^2^ = 0.834, *F*_1,45_ = 149.711, *P* = 9.323 × 10^−16^); (H) *J*_max_ versus *G*_m_ (regression *R*^2^ = 0.818, *F*_1,45_ = 136.184, *P* = 3.323 × 10^−15^); (I) *J*_max_ versus *G*_tot_ (regression *R*^2^ = 0.883, *F*_1,45_ = 161.692, *P* = 2.470 × 10^−16^).

Photosynthetic response curves to increased [CO_2_] (Fig. 2) performed *in situ* suggest that modification of the photosynthetic physiology with elevation occurred in concert with shifts in *G*_tot_. *Quercus spinosa* exhibited 46.5 and 76.1 % reductions in *V*_cmax_ and *J*_max_, respectively, that translated into 56 % reduction in the ratio of *J*_max_ to *V*_cmax_ (Table 2). In contrast, both *S. atopantha* and *R. dentatus* exhibited increased values of conductance to CO_2_ (Table 1) and corresponding increases in the physiological capacity to assimilate CO_2_ (Table 2). *Salix atopantha* and *R.dentatus*, respectively, showed 38.1 and 25.4 % increases in *V*_cmax_ alongside 52.8 and 34.7 % rises in *J*_max_ that did not significantly alter the *J*_max_ to *V*_cmax_ ratio of either species. The maximum rate of photosynthesis (*A*_max_), *V*_cmax_ and *J*_max_ all correlated to *G*_s_, *G*_m_ and *G*_tot_ (Fig. 3). However, at higher *G*_s_, *G*_m_ and *G*_tot_ values, photosynthetic capacity stabilizes and no longer increases, possibly due to physiological limitations to the rate of photosynthesis. Individuals of the annual herb *R. dentatus* at the higher elevation displayed the greatest values of *A*_max_, *V*_cmax_ and *J*_max_, while at the higher elevation, *Quercus* species exhibited the lowest levels of conductance and photosynthetic capacity to assimilate CO_2_ (Fig. 3).

**Table 2.** Photosynthetic parameters of the three species growing at lower and higher elevations (detailed in Table 1). Values of *A*_max_, *V*_cmax_ and *J*_max_ were obtained by fitting the Farquhar *et al*. (1980) model of leaf photosynthesis to five *A*/*P*_i_ response curves for each species at each elevation. Means of a parameter followed by the same letter were not statistically different using a one-way ANOVA (*P* > 0.05) with LSD *post hoc* test.

	*Q. spinosa*	*S. atopantha*	*R. dentatus*	*F*_5,30_	*P*-value
*A*_max_ (μmol m^−2^ s^−1^)					
Low elevation	23.31 ± 1.33 b	28.52 ± 1.18 c	32.50 ± 0.95 d	171.800	3.934 × 10^−21^
High elevation	7.75 ± 0.55 a	46.02 ± 1.23 d	55.45 ± 1.47 e		
*V*_cmax_ (μmol m^−2^ s^−1^)					
Low elevation	58.26 ± 2.42 b	58.56 ± 2.22 b	81.25 ± 2.87 c	72.301	8.123 × 10^−6^
High elevation	31.73 ± 1.66 a	80.85 ± 2.49 c	101.90 ± 3.86 c		
*J*_max_ (μmol m^−2^ s^−1^)					
Low elevation	236.70 ± 13.06 b	249.49 ± 13.40 b	334.37 ± 19.66 c	79.564	2.168 × 10^−16^
High elevation	56.65 ± 4.36 a	381.31 ± 6.49 c	450.36 ± 21.61 d		
*J*_max_/*V*_cmax_					
Low elevation	4.09 ± 0.24 b	4.25 ± 0.10 b	4.10 ± 0.15 b	41.107	1.533 × 10^−12^
High elevation	1.80 ± 0.13 a	4.73 ± 0.09 b	4.42 ± 0.16 b

**Figure 4.**
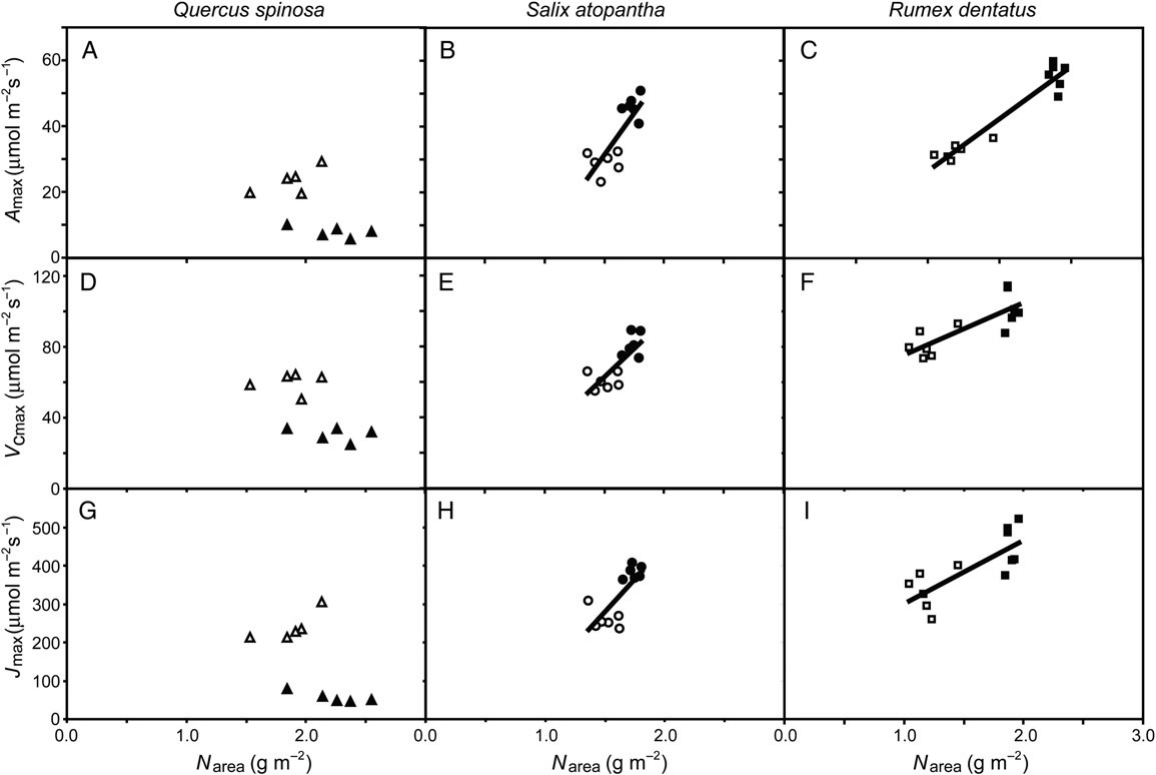
Relationship between foliar nitrogen concentration (N_area_) and parameters of physiological photosynthetic capacity (*A*_max_, *V*_cmax_ and *J*_max_) of *Q. spinosa* (upward triangles), *S. atopantha* (circles) and *R. dentatus* (squares) grown at high elevations of 2500 m (open symbols) and 3500 m (filled symbols) a.s.l. *Quercus spinosa*: (A) *A*_max_ versus N_area_ (regression *R*^2^ = 0.285, *F*_1,45_ = 3.195, *P* = 0.112); (D) *V*_cmax_ versus N_area_ (regression *R*^2^ = 0.394, *F*_1,45_ = 5.197, *P* = 0.0521) and (G) *J*_max_ versus N_area_ (regression *R*^2^ = 0.310, *F*_1,45_ = 3.589, *P* = 0.0948). *Salix atopantha*: (B) *A*_max_ versus N_area_ (regression *R*^2^ = 0.633, *F*_1,45_ = 17.216, *P* = 0.00198); (E) *V*_cmax_ versus N_area_ (regression *R*^2^ = 0.604, *F*_1,45_ = 15.260, *P* = 0.00293) and (H) *J*_max_ versus N_area_ (regression *R*^2^ = 0.550, *F*_1,45_ = 12.236, *P* = 0.00575). *Rumex dentatus*: (C) *A*_max_ versus N_area_ (regression *R*^2^ = 0.919, *F*_1,45_ = 113.380, *P* = 8.914 × 10^−07^); (F) *V*_cmax_ versus N_area_ (regression *R*^2^ = 0.639, *F*_1,45_ = 17.7124, *P* = 0.00180) and (I) *J*_max_ versus N_area_ (regression *R*^2^ = 0.596, *F*_1,45_ = 14.733, *P* = 0.00327).

The photosynthetic capacity of a leaf is generally related to the concentration of nitrogen within the foliage (Evans 1989). Both *S. atopantha* and *R. dentatus* showed increases in leaf nitrogen alongside *A*_max_, *V*_cmax_ and *J*_max_ with elevation (Fig. 4). Despite exhibiting reduced photosynthetic capacity and a 57 % increase in nitrogen concentration at higher elevations, *Q. spinosa* showed no significant relationship between leaf nitrogen and *A*_max_, *V*_cmax_ and *J*_max_. The PNUE values of *S. atopantha* and *R. dentatus* increased by 79.4 and 16.7 % at the higher elevation, while *Q. spinosa* showed a 69.2 % reduction of PNUE at 3500 m a.s.l. All three species showed increased foliar nitrogen concentration at the higher elevation, but this did not correspond to an increase in SLA (Table 3). While the evergreen *Q. spinosa* and annual herb *R. dentatus* showed respective reductions of 13.0 and 31.5 % in SLA at higher elevation, the SLA of *S. atopantha* was relatively unchanged. Growth at the higher elevation of 3500 m resulted in significant increases of 5.3–10.2 % in the foliar δ^13^C of all three species. The δ^13^C values of the plants from 2500 m a.s.l. were significantly enriched in the heavier ^13^C isotope relative to C3 plants growing at sea level (approximately +1–2‰) (Körner *et al*. 1988), and this enrichment in ^13^C became more pronounced at 3500 m a.s.l. (approximately +3–4‰).

**Table 3.** Leaf carbon isotopic composition (δ^13^C), SLA, leaf nitrogen concentration per unit mass (N_mass_), leaf nitrogen concentration per unit area (N_area_) and leaf PNUE of the three plants growing at lower and higher elevations (elevations detailed in Table 1). Means of a parameter followed by the same letter were not statistically different using a one-way ANOVA (*P* > 0.05) with LSD *post hoc* test.

	*Q. spinosa*	*S. atopantha*	*R. dentatus*	*F*_5,30_	*P*-value
δ^13^C (‰)					
Low elevation	−28.04 ± 0.03 c	−29.28 ± 0.19 b	−30.63 ± 0.09 a	35.525	9.894 × 10^−12^
High elevation	−26.23 ± 0.34 d	−27.74 ± 0.06 c	−27.52 ± 0.18 c		
SLA (cm^2^ g^−1^)					
Low elevation	92.82 ± 2.01 b	145.95 ± 5.68 c	288.31 ± 11.34 d	29.869	2.109 × 10^−10^
High elevation	80.71 ± 3.22 a	145.68 ± 2.68 c	197.59 ± 2.24 e		
N_area_ (g m^−2^)					
Low elevation	1.87 ± 0.09 c	1.51 ± 0.04 b	1.21 ± 0.05 a	147.774	3.182 × 10^−19^
High elevation	2.23 ± 0.11 d	1.74 ± 0.02 c	1.90 ± 0.02 cd		
PNUE (μmol mol^−1^ s^−1^)					
Low elevation	63.65 ± 4.64 b	65.56 ± 2.44 b	134.82 ± 1.71 d	210.276	2.101 × 10^−22^
High elevation	19.61 ± 1.65 a	117.64 ± 8.36 c	157.38 ± 4.25 d

**Figure 5.**
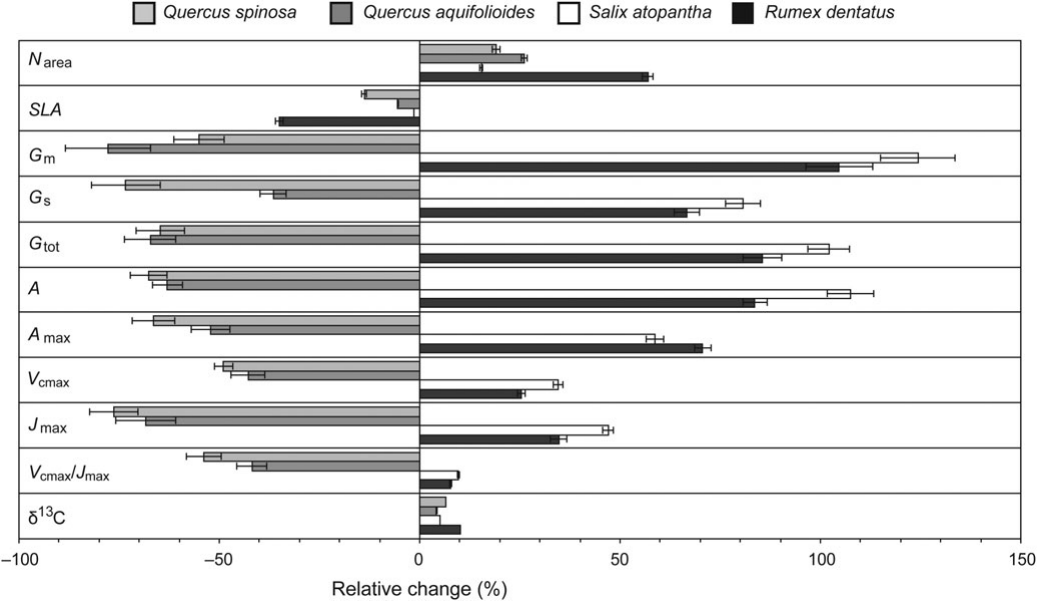
Relative effect of an increase in elevation from 2500 to 3500 m a.s.l. on photosynthetic, morphological and compositional characteristics of *Q. spinosa*, *S. atopantha* and *R. dentatus* from this study and *Q. aquifolioides* from the study of Feng *et al*. (2013). Error bars indicate 1 SE.

The results of this study have indicated the coordination of photosynthetic, gas exchange and morphological foliar responses to growth at high elevations of 2500 and 3500 m a.s.l. Nonetheless, two generally divergent responses to increased elevation become apparent from the study between the evergreen *Quercus* species with leaf lifespans of 1–3 years, and *S. atopantha* and *R. dentatus* that possess foliage with a leaf lifespan of <9 months. To illustrate these contrasting leaf responses to increased elevation, the relative changes of the physiological, morphological and compositional responses were plotted in Fig. 5. These suggest that the sclerophyllous *Quercus* species generally reduce conductance to CO_2_ and photosynthetic capacity with an increase in elevation from 2500 to 3500 m a.s.l., while the shorter-lived foliage of *S. atopantha* and *R. dentatus* showed the opposite response.

## Discussion

The results of our study have demonstrated contrasting physiological and morphological plasticity responses to an increase in elevation from 2500 to 3500 m a.s.l. in three Chinese montane species. *Quercus spinosa* and *Q. aquifolioides* possess robust sclerophyllous evergreen foliage with an average leaf lifespan >1 year. These *Quercus* species generally showed reductions in leaf gas exchange and photosynthetic capacity at the higher elevation. In contrast, the shorter-lived mesophytic foliage of *S. atopantha* and *R. dentatus* exhibited increases in conductance to CO_2_ and the capacity of photosynthetic physiology at the higher elevation (Fig. 5). This may suggest that the response of the plant species analysed to increased elevation was associated with growth habit and leaf economic traits (Milla and Reich 2011).

A rise in elevation confers a number of challenges to photosynthesis such as reduced *p*CO_2_ and temperature (Körner 2007). Despite the contrasting responses in terms of conductance to CO_2_ and photosynthetic physiology observed among different species in this study, increased elevation did result in more positive foliar δ^13^C, greater nitrogen concentration, enhanced *R*_n_ and reduced SLA in all species. At the same latitude, the δ^13^C of CO_2_ within the atmosphere is generally lower at high elevations (Trolier *et al*. 1996). The increased δ^13^C observed in all four species growing at 3500 m reflects the lower availability of CO_2_ reducing discrimination against ^13^C (Farquhar *et al*. 1989), despite the heavier isotope forming a reduced proportion of CO_2_ at the higher elevation (Trolier *et al*. 1996). The δ^13^C of a leaf is frequently affected by *G*_s_ and is indicative of the water use efficiency of a plant (Farquhar and Richards 1984; Farquhar *et al*. 1989). However, δ^13^C increases in all three species, despite *S. atopantha* and *R. dentatus* exhibiting increased *G*_s_ at the higher elevation (Fig. 1), suggest that at elevations >2500 m, factors other than *G*_s_ may influence carbon isotope discrimination. The increase in δ^13^C values of plants with elevation is commonly considered to reflect reduced *P*_i_/*P*_a_ ratios. However, due to modification of *G*_tot_, *V*_cmax_ and *J*_max_ with elevation, none of the species analysed in this study exhibited reduced *P*_i_/*P*_a_ ratios at the higher elevation (Table 1). Nonetheless, increased foliar δ^13^C observed in this study may indicate longer-term reductions in the *P*_i_/*P*_a_ ratio of the three species that was not apparent during the comparatively short duration of the gas exchange measurements. However, the δ^13^C values of the species analysed in this study are at the lower end of the range of values exhibited by plant species adapted to growth at high elevations (i.e. >2500 m) (Körner *et al*. 1988), possibly indicating that the combination of reduced *P*_i_/*P*_a_ ratios alongside the decreased availability of CO_2_ may induce lower δ^13^C values in other species.

The concentration of nitrogen per unit leaf area of the four species increased on average 29.4 % with a rise in elevation from 2500 to 3500 m a.s.l. (Fig. 5). Foliar nitrogen content and SLA are positively related to *A* (Poorter *et al*. 1990; Terashima *et al*. 2011). Point gas exchange measurements of *A* were taken under identical conditions of temperature, light and [CO_2_] at both 2500 and 3500 m a.s.l. This showed increased *A* at the higher elevation in the two species with short-lived foliage and lower *A* in the evergreen *Quercus* leaves. The greater values of *A* at the higher elevation in the two species where *G*_s_ and *G*_m_ rose with elevation may suggest that temperature plays a major role in limiting photosynthesis, thus affecting δ^13^C and determining the response of plants to elevation. In plants adapted to cool climates, leaves developed under low temperatures exhibited higher *V*_cmax_ and *J*_max_ values (Bunce 2000). However, despite exhibiting an increase in N_area_ at the higher elevation, the two *Quercus* species showed reductions in *A*, *V*_cmax_ and *J*_max_ at the greater elevation; this suggests that the greater N_area_ observed in *Q. spinosa* and *Q. aquifolioides* at the higher elevation was not associated with increased allocation of nitrogen to photosynthetic physiology (cf. Shi *et al*. 2006), or that diffusive limitations imposed by reduced *G*_s_ and *G*_m_ constrain any effect of increased allocation of nitrogen to RubisCO on *A* (e.g. Centritto *et al*. 2003; Loreto and Centritto 2008). The decrease in the *J*_max_ to *V*_cmax_ ratio observed in *Q. spinosa* is indicative of reduced allocation of nitrogen into light harvesting activities (Wullschleger 1993), possibly due to increased levels of radiation at the higher elevation. The greater values of *A* evident at higher elevations from the point measurements of leaf gas exchange at identical light and temperature may reflect the enhanced photosynthetic capacity and *G*_tot_ of *S. atopantha* and *R. dentatus*. This enhancement of *A* at higher elevations would likely not be evident under ambient growth conditions where temperatures and high incident radiation would not be conducive to photosynthesis (Fujimura *et al*. 2010). Woody species with thick sclerophyllous leaves generally have lower levels of *G*_tot_ than deciduous species that exhibit higher SLA values (Centritto *et al*. 2011). This lower conductance to CO_2_ reduces *P*_c_ levels and as a result constrains photosynthetic rates (Flexas *et al*. 2013; Lauteri *et al*. 2014). Any increase in the availability of CO_2_ will disproportionately affect evergreen species with low SLA values due to this limitation on transport of CO_2_ to the chloroplast envelope (Centritto *et al*. 2011; Niinemets *et al*. 2011). Future rises in atmospheric [CO_2_] would, therefore, most likely favour evergreen species growing at high elevations such as *Q. spinosa* rather than those plants with short leaf lifespans such as *S. atopantha* that exhibit increased *G*_tot_ with elevation (Table 1).

The divergent responses between the species may be due to the selective pressures associated with long- and short-lived foliage (Reich *et al*. 1991). Higher elevations experience factors such as increased incident radiation, drier air and stronger winds that increase the leaf to air vapour pressure deficit and the transpirative demand per unit leaf area (Körner and Diemer 1987; Körner 2007; Kouwenberg *et al*. 2007). Evergreen species such as *Q. spinosa* and *Q. aquifolioides* maintain leaves during periods that are not conducive to photosynthesis; this may necessitate a reduction in levels of *G*_s_ to prevent desiccation. In contrast, at higher elevations, the species with short-lived foliage can more fully exploit episodes where conditions are favourable to photosynthesis through enhanced levels of *G*_s_ and *G*_m_ at higher elevations before dispensing with leaves when growth conditions deteriorate and excessive transpirative and carbon balance costs are incurred. This behaviour does, however, incur costs in terms of the replacement of foliage each year, accounting for the lower SLA values of *S. atopantha* and *R. dentatus* in comparison with the two species of *Quercus* (Table 3). However, the construction costs per leaf are lower as investment in robust foliage capable of tolerating physical abrasion in a high energy windy environment (Wilson 1984) or physiological protection from high levels of harmful radiation over long periods of time is not required for leaves with a short leaf lifespan (Venema *et al*. 2000).

The number of mitochondria per unit leaf area increases with elevation (Miroslavov and Kravkina 1991), accounting for the increased *R*_n_ values at higher elevations observed in this (Table 2) and other studies (Ledig and Korbobo 1983; Shi *et al*. 2006; Feng *et al*. 2013). This increased *R*_n_ may be associated with lower partial pressures reducing the availability of oxygen (Crawford 1992), the increased respiratory demand required to support an enhanced photosynthetic physiology (Shi *et al*. 2006) or reduced ambient growth temperatures that result in higher *R*_n_ values when *R*_n_ is determined at the same leaf temperature (Atkin and Tjoelker 2003). When grown in a common garden study, 11 plant species collected from high elevations exhibited enhanced respiration at high temperatures relative to individuals of the same species that originated at low elevations. However, when temperature was reduced, *R*_n_ values were identical between individuals from low and high elevations (Larigauderie and Körner 1995). This may suggest that respiratory adaptation to growth at high elevations is due to the lower temperatures experienced at elevation. Temperature is likely a major factor in shaping plant photosynthetic responses to growth at high elevations. The reduction in temperature associated with an increase in elevation from 2500 to 3500 m a.s.l. will result in lower photosynthetic activity (Brooks and Farquhar 1985). The two species with short leaf lifespans will be able to shed their foliage during winter, whereas the foliage of the evergreen *Quercus* species will have to withstand the months with the lowest temperatures. This will necessitate a degree of morphological (Cordell *et al*. 1998) and physiological (Körner and Diemer 1987) tolerance to low temperatures in the evergreen species (Öquist and Huner 2003) and may account for the generally higher values of *R*_n_ observed in the leaves of *Q. spinosa* at 3500 m a.s.l. Furthermore, the effect of the decline in temperature associated with increased elevation is more apparent in trees that are directly affected by atmospheric circulation than shrub or herb layer plants that are smaller and generally sheltered, and as a result under radiation can maintain a significantly higher temperature than the surrounding trees (Körner 2007). This differential effect of temperature changes at high elevation may contribute to the increase in *G*_tot_ and photosynthetic capacity observed in *S. atopantha* and *R. dentatus*.

## Conclusions

The three plant species analysed in this study exhibited significant physiological and morphological plasticity to enable growth at 2500 and 3500 m a.s.l., an elevational gradient equivalent to a 5.5 °C decline in temperature and 11.2 % reduction in *p*CO_2_. The results of this study suggest that the physiological and morphological adaptations required for growth at high elevations may be associated with plant growth habit and leaf economics. Consistent with previous studies, all three species showed increased δ^13^C, *R*_n_ and leaf nitrogen at the higher elevation. However, critical differences in the photosynthetic and leaf gas exchange response to elevation were observed between the plants. The evergreen, *Q. spinosa*, showed reduced conductance to CO_2_ and diminished levels of *V*_cmax_ and *J*_max_ at the higher elevation. In contrast, *S. atopantha* and *R. dentatus* with a leaf lifespan of <9 months exhibited increased *G*_tot_ and enhanced photosynthetic capacity to fix CO_2_ at 3500 m. The selective pressures exerted by an increase in elevation may act differently dependent on the leaf lifespan of a species. Those species with short leaf lifespans can more fully exploit favourable growth conditions through increased conductance to CO_2_ and *A* before shedding foliage as conditions become less conducive to photosynthesis. Whereas evergreen species need to invest in physically robust leaves and physiological protective mechanisms to endure unfavourable conditions, this may necessitate a decrease in *G*_s_ to reduce water loss associated with the higher transpirative demands at higher elevations due to increased wind and radiation. Climate change may affect the plant species that compose high-elevation ecosystems differently depending on leaf economic traits as increased *p*CO_2_ is likely to benefit evergreen species with thick sclerophyllous leaves to a greater extent than deciduous species.

## Sources of Funding

This work was supported by National Natural Science Foundation of China (No. 30771718), Project in the National Science and Technology Pillar Program during the Twelfth Five-year Plan Period (No. 2012BAD22B0102); the Ministero dell'Istruzione, dell'Università e della Ricerca of Italy: PRIN 2010–2011 ‘PRO-ROOT’ and Progetto Premiale 2012 ‘Aqua’ and a Marie Curie IEF (2010-275626).

## Contributions by the Authors

Z.S. and M.C. designed the experiment. Z.S., Q.F. and R.C. conducted the measurements. M.H. and M.C. analysed the data and wrote the manuscript.

## Conflict of Interest Statement

None declared.

## References

[PLV115C1] Allen DJ, Ort DR 2001 Impacts of chilling temperatures on photosynthesis in warm-climate plants. Trends in Plant Science 6:36–42. 10.1016/S1360-1385(00)01808-2 11164376

[PLV115C2] Atkin OK, Tjoelker MG 2003 Thermal acclimation and the dynamic response of plant respiration to temperature. Trends in Plant Science 8:343–351. 10.1016/S1360-1385(03)00136-5 12878019

[PLV115C3] Bai Y-J, Chen L-Q, Ranhotra PS, Wang Q, Wang Y-F, Li C-S 2015 Reconstructing atmospheric CO_2_ during the Plio–Pleistocene transition by fossil *Typha*. Global Change Biology 21:874–881. 10.1111/gcb.12670 24990109

[PLV115C4] Barry RG 2013 Mountain weather and climate. Oxford: Routledge.

[PLV115C5] Brooks A, Farquhar GD 1985 Effect of temperature on the CO_2_/O_2_ specificity of ribulose-1,5-bisphosphate carboxylase/oxygenase and the rate of respiration in the light. Planta 165:397–406. 10.1007/BF00392238 24241146

[PLV115C6] Bunce JA 2000 Acclimation of photosynthesis to temperature in eight cool and warm climate herbaceous C_3_ species: temperature dependence of parameters of a biochemical photosynthesis model. Photosynthesis Research 63:59–67. 10.1023/A:1006325724086 16252165

[PLV115C7] Centritto M, Loreto F, Chartzoulakis K 2003 The use of low [CO_2_] to estimate diffusional and non-diffusional limitations of photosynthetic capacity of salt-stressed olive saplings. Plant, Cell and Environment 26:585–594. 10.1046/j.1365-3040.2003.00993.x

[PLV115C8] Centritto M, Lauteri M, Monteverdi MC, Serraj R 2009 Leaf gas exchange, carbon isotope discrimination, and grain yield in contrasting rice genotypes subjected to water deficits during the reproductive stage. Journal of Experimental Botany 60:2325–2339. 10.1093/jxb/erp123 19443613

[PLV115C9] Centritto M, Tognetti R, Leitgeb E, Střelcová K, Cohen S 2011 Above ground processes—anticipating climate change influences. In: Bredemeier M, Cohen S, Godbold DL, Lode E, Pichler V, Schleppi P, eds. Forest management and the water cycle: an ecosystem-based approach. London: Springer.

[PLV115C10] Chen-Fu F, Skvortsov AK 1998 Validation of Hao's new Chinese taxa in *Salix* (Salicaceae). Novon 8:467–470. 10.2307/3391877

[PLV115C11] Cordell S, Goldstein G, Mueller-Dombois D, Webb D, Vitousek PM 1998 Physiological and morphological variation in *Metrosideros polymorpha*, a dominant Hawaiian tree species, along an altitudinal gradient: the role of phenotypic plasticity. Oecologia 113:188–196. 10.1007/s004420050367 28308196

[PLV115C12] Crawford R 1992 Oxygen availability as an ecological limit to plant distribution. Advances in Ecological Research 23:93–185.

[PLV115C13] Evans JR 1989 Photosynthesis and nitrogen relationships in leaves of C_3_ plants. Oecologia 78:9–19. 10.1007/BF00377192 28311896

[PLV115C14] Farquhar GD, Richards RA 1984 Isotopic composition of plant carbon correlates with water-use efficiency of wheat genotypes. Functional Plant Biology 11:539–552.

[PLV115C15] Farquhar GD, Caemmerer S, Berry JA 1980 A biochemical model of photosynthetic CO_2_ assimilation in leaves of C_3_ species. Planta 149:78–90. 10.1007/BF00386231 24306196

[PLV115C16] Farquhar GD, Ehleringer JR, Hubick KT 1989 Carbon isotope discrimination and photosynthesis. Annual Review of Plant Physiology and Plant Molecular Biology 40:503–537. 10.1146/annurev.pp.40.060189.002443

[PLV115C17] Feng Q, Centritto M, Cheng R, Liu S, Shi Z 2013 Leaf functional trait responses of *Quercus aquifolioides* to high elevations. International Journal of Agriculture and Biology 15:69–75.

[PLV115C18] Flexas J, Diaz-Espejo A, Galmés J, Kaldenhoff R, Medrano H, Ribas-Carbo M 2007 Rapid variations of mesophyll conductance in response to changes in CO_2_ concentration around leaves. Plant, Cell and Environment 30:1284–1298. 10.1111/j.1365-3040.2007.01700.x 17727418

[PLV115C19] Flexas J, Niinemets Ü, Gallé A, Barbour MM, Centritto M, Diaz-Espejo A, Douthe C, Galmés J, Ribas-Carbo M, Rodriguez PL, Rosselló F, Soolanayakanahally R, Tomas M, Wright IJ, Farquhar GD, Medrano H 2013 Diffusional conductances to CO_2_ as a target for increasing photosynthesis and photosynthetic water-use efficiency. Photosynthesis Research 117:45–59. 10.1007/s11120-013-9844-z 23670217

[PLV115C20] Friend AD, Woodward FI, Switsur VR 1989 Field measurements of photosynthesis, stomatal conductance, leaf nitrogen and δ^13^C along altitudinal gradients in Scotland. Functional Ecology 3:117–122. 10.2307/2389682

[PLV115C21] Friend DJC, Pomeroy ME 1970 Changes in cell size and number associated with the effects of light intensity and temperature on the leaf morphology of wheat. Canadian Journal of Botany 48:85–90. 10.1139/b70-011

[PLV115C22] Fryer MJ, Andrews JR, Oxborough K, Blowers DA, Baker NR 1998 Relationship between CO_2_ assimilation, photosynthetic electron transport, and active O_2_ metabolism in leaves of maize in the field during periods of low temperature. Plant Physiology 116:571–580. 10.1104/pp.116.2.571 9490760 PMC35114

[PLV115C23] Fujimura S, Shi P, Iwama K, Zhang X, Gopal J, Jitsuyama Y 2010 Effect of altitude on the response of net photosynthetic rate to carbon dioxide increase by spring wheat. Plant Production Science 13:141–149. 10.1626/pps.13.141

[PLV115C24] Gale J 1972a Availability of carbon dioxide for photosynthesis at high altitudes: theoretical considerations. Ecology 53:494–497. 10.2307/1934239

[PLV115C25] Gale J 1972b Elevation and transpiration: some theoretical considerations with special reference to Mediterranean-type climate. Journal of Applied Ecology 9:691–702. 10.2307/2401898

[PLV115C26] Gale J 1973 Experimental evidence for the effect of barometric pressure on photosynthesis and transpiration. In: Slatyer RO, ed. Ecology and conservation: plant response to climatic factors. Proceedings of the Uppsala Symposium. Paris: UNESCO.

[PLV115C27] Galmes J, Flexas J, Keys AJ, Cifre J, Mitchell RAC, Madgwick PJ, Haslam RP, Medrano H, Parry MAJ 2005 Rubisco specificity factor tends to be larger in plant species from drier habitats and in species with persistent leaves. Plant, Cell and Environment 28:571–579. 10.1111/j.1365-3040.2005.01300.x

[PLV115C28] Genty B, Briantais J-M, Baker NR 1989 The relationship between the quantum yield of photosynthetic electron transport and quenching of chlorophyll fluorescence. Biochimica et Biophysica Acta (BBA) - General Subjects 990:87–92. 10.1016/S0304-4165(89)80016-9

[PLV115C29] Gilbert ME, Pou A, Zwieniecki MA, Holbrook NM 2012 On measuring the response of mesophyll conductance to carbon dioxide with the variable J method. Journal of Experimental Botany 63:413–425. 10.1093/jxb/err288 21914657 PMC3245476

[PLV115C30] Harley PC, Loreto F, Di Marco G, Sharkey TD 1992 Theoretical considerations when estimating the mesophyll conductance to CO_2_ flux by analysis of the response of photosynthesis to CO_2_. Plant Physiology 98:1429–1436. 10.1104/pp.98.4.1429 16668811 PMC1080368

[PLV115C31] Haworth M, Raschi A 2014 An assessment of the use of epidermal micro-morphological features to estimate leaf economics of Late Triassic-Early Jurassic fossil Ginkgoales. Review of Palaeobotany and Palynology 205:1–8. 10.1016/j.revpalbo.2014.02.007

[PLV115C32] Haworth M, Elliott-Kingston C, Mcelwain JC 2011 Stomatal control as a driver of plant evolution. Journal of Experimental Botany 62:2419–2423. 10.1093/jxb/err086 21576397

[PLV115C33] Haworth M, Killi D, Materassi A, Raschi A 2015 Coordination of stomatal physiological behavior and morphology with carbon dioxide determines stomatal control. American Journal of Botany 102:677–688. 10.3732/ajb.1400508 26022482

[PLV115C34] Hikosaka K, Nagamatsu D, Ishii HS, Hirose T 2002 Photosynthesis-nitrogen relationships in species at different altitudes on Mount Kinabalu, Malaysia. Ecological Research 17:305–313. 10.1046/j.1440-1703.2002.00490.x

[PLV115C35] Hovenden MJ, Vander Schoor JK 2004 Nature vs nurture in the leaf morphology of Southern beech, *Nothofagus cunninghamii* (Nothofagaceae). New Phytologist 161:585–594. 10.1046/j.1469-8137.2003.00931.x 33873506

[PLV115C36] Hu J-J, Xing Y-W, Turkington R, Jacques FMB, Su T, Huang Y-J, Zhou Z-K 2015 A new positive relationship between pCO_2_ and stomatal frequency in *Quercus guyavifolia* (Fagaceae): a potential proxy for palaeo-CO_2_ levels. Annals of Botany 115:777–788. 25681824 10.1093/aob/mcv007 PMC4373289

[PLV115C37] Hu L, Wang Z, Huang B 2010 Diffusion limitations and metabolic factors associated with inhibition and recovery of photosynthesis from drought stress in a C_3_ perennial grass species. Physiologia Plantarum 139:93–106. 10.1111/j.1399-3054.2010.01350.x 20070869

[PLV115C38] Kogami H, Hanba YT, Kibe T, Terashima I, Masuzawa T 2001 CO_2_ transfer conductance, leaf structure and carbon isotope composition of *Polygonum cuspidatum* leaves from low and high altitudes. Plant, Cell and Environment 24:529–538. 10.1046/j.1365-3040.2001.00696.x

[PLV115C39] Körner C 2007 The use of ‘altitude’ in ecological research. Trends in Ecology and Evolution 22:569–574. 10.1016/j.tree.2007.09.006 17988759

[PLV115C40] Körner C, Cochrane PM 1985 Stomatal responses and water relations of *Eucalyptus pauciflora* in summer along an elevational gradient. Oecologia 66:443–455. 10.1007/BF00378313 28310877

[PLV115C41] Körner C, Diemer M 1987 In situ photosynthetic responses to light, temperature and carbon dioxide in herbaceous plants from low and high altitude. Functional Ecology 1:179–194. 10.2307/2389420

[PLV115C42] Körner C, Scheel JA, Bauer H 1979 Maximum leaf diffusive conductance in vascular plants. Photosynthetica 13:45–82.

[PLV115C43] Körner C, Bannister P, Mark AF 1986 Altitudinal variation in stomatal conductance, nitrogen content and leaf anatomy in different plant life forms in New Zealand. Oecologia 69:577–588. 10.1007/BF00410366 28311619

[PLV115C44] Körner C, Farquhar GD, Roksandic Z 1988 A global survey of carbon isotope discrimination in plants from high altitude. Oecologia 74:623–632. 10.1007/BF00380063 28311772

[PLV115C45] Kouwenberg LLR, Kürschner WM, Mcelwain JC 2007 Stomatal frequency change over altitudinal gradients: prospects for paleoaltimetry. Reviews in Mineralogy and Geochemistry 66:215–241. 10.2138/rmg.2007.66.9

[PLV115C46] Kumar N, Kumar S, Ahuja PS 2005 Photosynthetic characteristics of *Hordeum*, *Triticum*, *Rumex*, and *Trifolium* species at contrasting altitudes. Photosynthetica 43:195–201. 10.1007/s11099-005-0033-y

[PLV115C47] Larigauderie A, Körner C 1995 Acclimation of leaf dark respiration to temperature in alpine and lowland plant species. Annals of Botany 76:245–252. 10.1006/anbo.1995.1093

[PLV115C48] Lauteri M, Haworth M, Serraj R, Monteverdi MC, Centritto M 2014 Photosynthetic diffusional constraints affect yield in drought stressed rice cultivars during flowering. PLoS ONE 9:e109054 10.1371/journal.pone.0109054 25275452 PMC4183539

[PLV115C49] Ledig FT, Korbobo DR 1983 Adaptation of sugar maple populations along altitudinal gradients: photosynthesis, respiration, and specific leaf weight. American Journal of Botany 70:256–265. 10.2307/2443271

[PLV115C50] Li C, Zhang X, Liu X, Luukkanen A, Berninger F 2006 Leaf morphological and physiological responses of *Quercus aquifolioides* along an altitudinal gradient. Silva Fennica 40:5–13.

[PLV115C51] Li Y, Yang D, Xiang S, Li G 2013 Different responses in leaf pigments and leaf mass per area to altitude between evergreen and deciduous woody species. Australian Journal of Botany 61:424–435. 10.1071/BT13022

[PLV115C52] Loreto F, Centritto M 2008 Leaf carbon assimilation in a water-limited world. Plant Biosystems 142:154–161. 10.1080/11263500701872937

[PLV115C53] Loreto F, Di Marco G, Tricoli D, Sharkey TD 1994 Measurements of mesophyll conductance, photosynthetic electron transport and alternative electron sinks of field grown wheat leaves. Photosynthesis Research 41:397–403. 10.1007/BF02183042 24310154

[PLV115C54] Loreto F, Tsonev T, Centritto M 2009 The impact of blue light on leaf mesophyll conductance. Journal of Experimental Botany 60:2283–2290. 10.1093/jxb/erp112 19395388

[PLV115C55] Milla R, Reich PB 2011 Multi-trait interactions, not phylogeny, fine-tune leaf size reduction with increasing altitude. Annals of Botany 107:455–465. 10.1093/aob/mcq261 21199835 PMC3043936

[PLV115C56] Miroslavov EA, Kravkina IM 1991 Comparative analysis of chloroplasts and mitochondria in leaf chlorenchyma from mountain plants grown at different altitudes. Annals of Botany 68:195–200.

[PLV115C57] Niinemets Ü 2001 Global-scale climatic controls of leaf dry mass per area, density, and thickness in trees and shrubs. Ecology 82:453–469. 10.1890/0012-9658(2001)082[0453:GSCCOL]2.0.CO;2

[PLV115C58] Niinemets Ü, Díaz-Espejo A, Flexas J, Galmés J, Warren CR 2009 Role of mesophyll diffusion conductance in constraining potential photosynthetic productivity in the field. Journal of Experimental Botany 60:2249–2270. 10.1093/jxb/erp036 19395391

[PLV115C59] Niinemets Ü, Flexas J, Peñuelas J 2011 Evergreens favored by higher responsiveness to increased CO_2_. Trends in Ecology and Evolution 26:136–142. 10.1016/j.tree.2010.12.012 21277042

[PLV115C60] Ögren E, Sundin U 1996 Photosynthetic responses to variable light: a comparison of species from contrasting habitats. Oecologia 106:18–27. 10.1007/BF00334403 28307153

[PLV115C61] Öquist G, Huner NPA 2003 Photosynthesis of overwintering evergreen plants. Annual Review of Plant Biology 54:329–355. 10.1146/annurev.arplant.54.072402.115741 14502994

[PLV115C62] Pan S, Liu C, Zhang W, Xu S, Wang N, Li Y, Gao J, Wang Y, Wang G 2013 The scaling relationships between leaf mass and leaf area of vascular plant species change with altitude. PLoS ONE 8:e76872 10.1371/journal.pone.0076872 24146938 PMC3795618

[PLV115C63] Poorter H, Remkes C, Lambers H 1990 Carbon and nitrogen economy of 24 wild species differing in relative growth rate. Plant Physiology 94:621–627. 10.1104/pp.94.2.621 16667757 PMC1077277

[PLV115C64] Poorter H, Niinemets Ü, Poorter L, Wright IJ, Villar R 2009 Causes and consequences of variation in leaf mass per area (LMA): a meta-analysis. New Phytologist 182:565–588. 10.1111/j.1469-8137.2009.02830.x 19434804

[PLV115C65] Reich PB, Uhl C, Walters MB, Ellsworth DS 1991 Leaf lifespan as a determinant of leaf structure and function among 23 Amazonian tree species. Oecologia 86:16–24. 10.1007/BF00317383 28313152

[PLV115C66] Rodeghiero M, Niinemets Ü, Cescatti A 2007 Major diffusion leaks of clamp-on leaf cuvettes still unaccounted: how erroneous are the estimates of Farquhar et al. model parameters? Plant, Cell and Environment 30:1006–1022. 10.1111/j.1365-3040.2007.001689.x 17617828

[PLV115C67] Shi B, Ming Y 1987 Wolong vegetation and resource plants. Chengdu, China: Sichuan Science and Technology Press.

[PLV115C68] Shi Z, Liu S, Liu X, Centritto M 2006 Altitudinal variation in photosynthetic capacity, diffusional conductance and δ^13^C of butterfly bush (*Buddleja davidii*) plants growing at high elevations. Physiologia Plantarum 128:722–731. 10.1111/j.1399-3054.2006.00805.x

[PLV115C69] Terashima I, Masuzawa T, Ohba H, Yokoi Y 1995 Is photosynthesis suppressed at higher elevations due to low CO_2_ pressure? Ecology 76:2663–2668. 10.2307/2265838

[PLV115C70] Terashima I, Hanba YT, Tholen D, Niinemets Ü 2011 Leaf functional anatomy in relation to photosynthesis. Plant Physiology 155:108–116. 10.1104/pp.110.165472 21075960 PMC3075775

[PLV115C71] Tholen D, Ethier G, Genty B, Pepin S, Zhu X-G 2012 Variable mesophyll conductance revisited: theoretical background and experimental implications. Plant, Cell and Environment 35:2087–2103. 10.1111/j.1365-3040.2012.02538.x 22590996

[PLV115C72] Trolier M, White JWC, Tans PP, Masarie KA, Gemery PA 1996 Monitoring the isotopic composition of atmospheric CO_2_: measurements from the NOAA Global Air Sampling Network. Journal of Geophysical Research: Atmospheres 101:25897–25916. 10.1029/96JD02363

[PLV115C73] Venema JH, Villerius L, Van Hasselt PR 2000 Effect of acclimation to suboptimal temperature on chilling-induced photodamage: comparison between a domestic and a high-altitude wild *Lycopersicon* species. Plant Science 152:153–163. 10.1016/S0168-9452(99)00228-9

[PLV115C74] Von Caemmerer S 2000 Biochemical models of leaf photosynthesis. Collingwood, Australia: CSIRO Publishing.

[PLV115C75] Walker BJ, Cousins AB 2013 Influence of temperature on measurements of the CO_2_ compensation point: differences between the Laisk and O_2_-exchange methods. Journal of Experimental Botany 64:1893–1905. 10.1093/jxb/ert058 23630324 PMC3638825

[PLV115C76] Wang R, Yu G, He N, Wang Q, Xia F, Zhao N, Xu Z, Ge J 2014 Elevation-related variation in leaf stomatal traits as a function of plant functional type: evidence from Changbai Mountain, China. PLoS ONE 9:e115395 10.1371/journal.pone.0115395 25517967 PMC4269444

[PLV115C77] Warren CR 2007 Stand aside stomata, another actor deserves centre stage: the forgotten role of the internal conductance to CO_2_ transfer. In: 14th International Congress of Photosynthesis Oxford University Press, Glasgow, Scotland. 10.1093/jxb/erm245 17975206

[PLV115C78] Williams DG, Black RA 1993 Phenotypic variation in contrasting temperature environments: growth and photosynthesis in *Pennisetum setaceum* from different altitudes on Hawaii. Functional Ecology 7:623–633. 10.2307/2390140

[PLV115C79] Wilson J 1984 Microscopic features of wind damage to leaves of *Acer pseudoplatanus* L. Annals of Botany 53:73–82.

[PLV115C80] Woodward FI 1986 Ecophysiological studies on the shrub *Vaccinium myrtillus* L. taken from a wide altitudinal range. Oecologia 70:580–586. 10.1007/BF00379908 28311503

[PLV115C81] Woodward FI, Bazzaz FA 1988 The responses of stomatal density to CO_2_ partial pressure. Journal of Experimental Botany 39:1771–1781. 10.1093/jxb/39.12.1771

[PLV115C82] Wu ZY, Raven PH, Hong DY 2011 Flora of China Volume 20-21. St. Louis: Beijing Science Press and Missouri Botanical Garden Press.

[PLV115C83] Wullschleger SD 1993 Biochemical limitations to carbon assimilation in C_3_ plants—a retrospective analysis of the *A*/*C_i_* curves from 109 species. Journal of Experimental Botany 44:907–920. 10.1093/jxb/44.5.907

